# Basic and clinical immunology – 3024. First evidence for epigenetic disruption in t-cells from children with food allergy

**DOI:** 10.1186/1939-4551-6-S1-P200

**Published:** 2013-04-23

**Authors:** David Jim Martino, S Eric Joo, Richard Saffery, Susan Prescott

**Affiliations:** 1 School of Paediatrics and Child Health, University of Western Australia, Australia; 2 Murdoch Childrens Research Institute, Australia

## Background

No studies to date have formally investigated the role of epigenetics in food allergy. Many believe that disruption in epigenetic marks may lead to loss of gene control in relevant immune pathways, and this may predispose to allergic disease. There is a growing body of evidence to suggest DNA methylation marks in key locus control regions are the primary regulators of naïve T-helper cell differentiation. To address these contemporary research questions, we undertook genome-wide methylation profiling of CD4+ T-cells harvested from children with and without food allergy, before and during the onset of disease. This data was analysed in the context of genome-wide expression data from the same cohort.

## Methods

Genome wide DNA methylation profiling of CD4+ cells from children with diagnosed food allergy (n=30) and non-allergic children (n=30) was undertaken at birth (neonatal cells) and 12 months (during onset). A comparative analysis of DNA methylation profiles was performed and this data was correlated with gene expression data and functional T-cell assays.

## Results

We report the first lines of evidence for epigenetic disruption in association with food allergy. 85 loci were differentially methylated between allergics and non-allergics after adjusting for age (Adj P-Value<0.05 Beta fold change >0.10), and genetic effects. This represented a change in the promoter methylation status in 25 unique genes involved in cellular response to stress, fatty acid beta-oxidation pathways, calcium-activated potassium channel activity, small molecule and vitamin metabolism. Approximately 40% of methylation changes occurred outside known gene-associated regions with unknown significance. An examination of the effects of SNPs on methylation profiles revealed HLA-DQB1 as differentially methylated between allergics and non-allergics, resulting in a quantitative change in gene expression.

## Conclusions

DNA methylation profiling of CD4+ cells reveals disruption of several epigenetic pathways that appear to be programmed into the T-cell compartment. Although a proxy marker, the methylation array has genotyping utility and suggests a novel role for SNPs in HLA-DQB1 in association with changes in methylation and gene expression.

